# Nuclear factor-kappa B localization and function within intrauterine tissues from term and preterm labor and cultured fetal membranes

**DOI:** 10.1186/1477-7827-8-8

**Published:** 2010-01-25

**Authors:** Sonali Vora, Asad Abbas, Chong J Kim, Taryn LS Summerfield, Juan P Kusanovic, Jay D Iams, Roberto Romero, Douglas A Kniss, William E Ackerman

**Affiliations:** 1 Laboratory of Perinatal Research, Department of Obstetrics & Gynecology, College of Medicine, The Ohio State University, Columbus, OH, USA; 2 Perinatology Research Branch, Eunice Kennedy Shriver National Institute of Child Health and Human Development, National Institutes of Health, Bethesda, MD, USA; 3 Department of Obstetrics and Gynecology, Wayne State University School of Medicine, Detroit, MI, USA; 4 Department of Pathology, Wayne State University School of Medicine, Detroit, MI, USA; 5 Division of Maternal-Fetal Medicine, Department of Obstetrics & Gynecology, College of Medicine, The Ohio State University, Columbus, OH, USA; 6 Department of Biomedical Engineering, College of Engineering, The Ohio State University, Columbus, OH, USA

## Abstract

**Background:**

The objective of this study was to quantify the nuclear localization and DNA binding activity of p65, the major transactivating nuclear factor-kappa B (NF-kappaB) subunit, in full-thickness fetal membranes (FM) and myometrium in the absence or presence of term or preterm labor.

**Methods:**

Paired full-thickness FM and myometrial samples were collected from women in the following cohorts: preterm no labor (PNL, N = 22), spontaneous preterm labor (PTL, N = 21), term no labor (TNL, N = 23), and spontaneous term labor (STL, N = 21). NF-kappaB p65 localization was assessed by immunohistochemistry, and DNA binding activity was evaluated using an enzyme-linked immunosorbent assay (ELISA)-based method.

**Results:**

Nuclear p65 labeling was rare in amnion and chorion, irrespective of clinical context. In decidua, nuclear p65 labeling was greater in the STL group relative to the TNL cohort, but there were no differences among the TNL, PTL, and PNL cohorts. In myometrium, diffuse p65 nuclear labeling was significantly associated with both term and preterm labor. There were no significant differences in ELISA-based p65 binding activity in amnion, choriodecidual, and myometrial specimens in the absence or presence of term labor. However, parallel experiments using cultured term fetal membranes demonstrated high levels of p65-like binding even the absence of cytokine stimulation, suggesting that this assay may be of limited value when applied to tissue specimens.

**Conclusions:**

These results suggest that the decidua is an important site of NF-kappaB regulation in fetal membranes, and that mechanisms other than cytoplasmic sequestration may limit NF-kappaB activation prior to term.

## Background

The precise molecular mechanisms that underpin the commencement of cervical effacement and dilatation, and robust, synchronous myometrial contractions resulting in expulsion of the fetus at term are still incompletely understood. Moreover, the untimely onset of labor prior to 37 weeks of gestation currently contributes to a 12.5% rate of preterm deliveries culminating in significant perinatal morbidity and mortality [1]. This actually represents a substantial increase over estimates from just a decade ago.

Despite the rather dismal epidemiological picture, there is now a nearly complete consensus that spontaneous preterm labor is initiated by a complex set of biochemical events that can be categorized as localized inflammation resulting from the untimely activation of the innate immune response within the intrauterine microenvironment [2,3]. To date, the chief proximate cause of these inflammatory sequelae is bacterial infections that colonize one or more compartments of the female urogenital tract and/or placenta and fetal membranes [4]. As further support for this notion, even systemic infections remote from the gravid uterus can incite inflammatory changes by eliciting immune activation and the release of circulating bioactive molecules such as cytokines, chemokines, and arachidonic acid metabolites [5]. Many of the most common of these biomediators are interleukins- 1β, 6, and 8 (IL- 1β, 6, 8), tumor necrosis factor-α (TNF-α), macrophage chemotactic peptide-1 (MCP-1), prostaglandins E_2 _and F_2α _(PGE_2 _and PGF_2α_), and nitric oxide (NO) [6-11]. IL-1β, IL-6 and TNF-α serve as the major immunomodulators, while IL-8 and MCP-1 are chemokines that recruit neutrophils and monocytes into sites of tissue inflammation [4]. PGE_2 _and PGF_2α _are potent bioactive lipids that stimulate immune functions such as vascular reactivity and permeability, and extracellular matrix remodeling [12].

Interestingly, each of these events is in some way or another governed by the canonical inflammatory transcription factors, nuclear factor-kappa B (NF-κB) [13,14]. That is, this DNA-binding protein either directly controls the genes encoding the cytokines and chemokines or it controls the genes that encode the rate-limiting enzymes that manufacture the mediators (PGs and NO) [15-17]. The NF-κB family of transcription factors is made up of at least five member proteins that reach back in evolution as far as the arthropods (e.g., fruit flies) and they appear to regulate nearly every aspect of modern immune responsiveness [18]. Thus, in our continuing attempts to identify the key players in the inflammatory events that underpin one of the most fundamental of reproductive events, the delivery of the offspring, we conducted *in vitro *and translational *in vivo *studies to link the localization and function of NF-κB in cells grown in culture (following stimulation with cytokines known to be present in preterm labor) with that in tissues collected in the absence or presence of human parturition.

## Methods

### Tissue collection and study design

Tissue biopsies from clinical samples were collected from women at the time of delivery following written informed consent and approval from the Institutional Review Boards (IRBs) of the Sotero del Rio Hospital, Santiago, Chile (an affiliate of the Pontificia Catholic University of Santiago, Chile), and the *Eunice Kennedy Shriver *National Institute of Child Health and Human Development, NIH, DHHS. Patients were classified in the following groups: preterm no labor (PNL, N = 22), spontaneous preterm labor (PTL, N = 21), term no labor (TNL, N = 23), and spontaneous term labor (STL, N = 21). Subjects delivered between gestational ages 26^0/0 ^- 36^6/7 ^weeks (preterm cohort; PNL or PTL) and 37^0/0 ^- 41^6/7 ^weeks (term cohort; TNL or STL). Paired full-thickness fetal membrane and myometrial biopsy samples were collected from patients who had undergone cesarean section for various medical conditions (hypertension, fetal growth restriction, macrosomia), or due to history of prior cesarean section, fetal malpresentation or an unfavorable cervix by clinical examination. Following delivery of the placenta, portions of reflected membranes were separated into amnion and choriodecidua and snap frozen in liquid N_2 _for subsequent analysis. In parallel, full-thickness membrane rolls were fixed in formalin in preparation for immunohistochemistry. For the collection of myometrial specimens, biopsies of the lower uterine segment were obtained at the time of cesarean section by dissecting a small portion of the uterine wall along the uterine incision prior to closure of the uterus. Myometrial specimens were either flash frozen in liquid N_2 _or fixed in formalin and paraffin embedded for subsequent analysis.

Selected demographic characteristics of the participants are presented in Table 1. In the preterm cohort, the PTL group tended to younger on average than the PNL group, but the gestational ages at delivery and birthweights were similar. In the term cohort, participants in the STL group were more likely to have been primigravid and nulliparous, which was anticipated given that subjects in the TNL group were more likely to have had a prior cesarean; other characteristics were similar. The most common indication for iatrogenic delivery in the PNL cohort was severe preeclampsia with abnormal Doppler velocimetry or hypertensive crisis. The prevalence of preeclampsia was 73% in the PNL cohort, compared with 5% in the PTL cohort.

**Table 1:** Selected maternal characteristics by cohort*

	Preterm Cohort			Term Cohort		
	PNL n = 22	PTL n = 21	P value	TNL n = 23	STL n = 21	P value
Maternal age (y)**	27.9 ± 6.7 (16-38)	23.4 ± 7.2 (13-37)	0.0399	29.3 ± 6.3 (19-41)	26.4 ± 6.9 (16-43)	N.S.
**Gestational age** at delivery (wk)**	33.1 ± 2.7 (27.3-36.3)	32.5 ± 3.0 (26.0-36.9)	N.S.	39.2 ± 1.1 (37.9-41.1)	39.5 ± 1.0 (37.6-41.1)	N.S.
Birthweight (g)**	1780 ± 520 (810-2810)	1980 ± 640 (910-3250)	N.S.	3524 ± 421 (2870-4750)	3572 ± 263 (3100-3960)	N.S.
**Gravidity**						
**1**	8	9	N.S.	0	7	0.0086
**2**	6	5		9	4	
**>2**	8	7		14	10	
**Parity**						
**0**	9	10	N.S.	1	8	0.0203
**1**	8	5		8	4	
**>1**	5	6		14	9	

* All specimens derived from these subjects were collected at Sotero del Rio Hospital, Santiago, Chile

** Values are given as mean ± standard deviation; the range is given in parentheses

N.S., not significant

### Primary amnion mesenchymal cell cultures

Fetal membranes used to prepare primary amnion mesenchymal cell (AMC) cultures were collected at the time of delivery from women who provided written informed consent under Biomedical IRB approval at The Ohio State University Medical Center. Primary cultures of human amnion mesenchymal cells were prepared from amnion membranes stripped of underlying chorionic tissue from placentas collected from women prior to the onset of labor at term as previously described [15]. Cells were cultured in high-glucose Dulbecco's modified Eagle's medium (DMEM; 25 mM glucose) supplemented with 10% fetal bovine serum (FBS), 2 mM L-glutamine, 1 mM sodium pyruvate, 50 μg/ml of gentamicin sulfate, and 0.5 μg/ml of amphotericin B at 37°C in a humidified incubator with 5% CO_2_. Upon reaching confluence, cultures were then treated in the absence or presence of 10 ng/ml of IL-1β or 20 ng/ml of TNF-α (both from R&D Systems, Minneapolis, MN) for 0-120 min, as described in the text and figure legends.

### Fetal membrane explant cultures

Full-thickness fetal membranes explant cultures were established from a limited number of placentas (N = 4) delivered at term by elective cesarean section following uncomplicated gestations at Hutzel Women's Hospital (Detroit, MI) with approval of the IRB of the *Eunice Kennedy Shriver *National Institute of Child Health and Human Development, NIH, DHHS. For each culture, intact portions of the reflected fetal membranes between the incision site and the placental disc were dissected into uniform 1 in^2 ^squares and placed in sterile PBS. These specimens were then rinsed free of clotted blood using sterile PBS. Full-thickness specimens, as well as bluntly separated amniotic and choriodecidual specimens, were cultured in parallel in serum-free RPMI media in a humidified incubator at 37°C in a 5% CO_2 _atmosphere. Following overnight incubations, cultures were then treated in the absence or presence of 10 ng/ml of IL-1β for 1-2 h, as described in the text and figure legends.

### Nuclear extraction

Nuclear extraction from cultured cells was performed using a nuclear extract kit according to the instructions of the manufacturer (Active Motif, Carlsbad, CA). Following treatments, cells were suspended in hypotonic buffer (20 mM HEPES, pH 7.5, 5 mM NaF, 10 μM Na_2_MoO_4_, and 0.1 mM EDTA) containing phosphatase inhibitors and incubated on ice for 15 minutes. Detergent (Nonidet P-40) was added to a final concentration of 0.5%, and incubation was continued for an additional 15 min. The lysate was then subjected to centrifugation (14,000 × *g *for 30 sec at 4°C) to pellet nuclei, and the supernatant was removed. The nuclear pellet was next extracted in a proprietary lysis buffer for 30 min on ice using a rotary shaker. Following centrifugation (14,000 × *g *for 30 min at 4°C), the supernatant containing the pure nuclear fraction was collected. The concentration of the nuclear extract was determined using the Bradford Assay [19] and measured at a 595 nm absorbance using the Dynex MLX microplate luminometer (Dynex Technologies, Chantally, VA).

For the isolation of nuclear proteins from fetal membrane explant cultures, pilot experiments were conducted in which the tissues were either minced finely and homogenized using a Dounce type homogenizer (protocol 1), or flash frozen and ground in liquid N_2 _using a mortar and pestle (protocol 2), prior to extraction. In each case, extraction was performed using the same reagents used for the cultured cells. Briefly, tissue was incubated in hypotonic buffer on ice and extracted using 0.5% Nonidet P-40. Following centrifugation, the resulting pellet (presumptive nuclear fraction) was suspended in a proprietary lysis buffer, and non-extracted cellular debris was removed by centrifugation.

For the isolation of nuclear proteins from flash frozen amnion, choriodecidua, and myometrial tissues, frozen samples were and ground in liquid N_2 _using a mortar and pestle prior to extraction using the protocol listed above.

### Transcription factor binding assay

The ELISA-based chemiluminescent NF-κB p65 binding assay kit was run according to the manufacturer's instructions (Active Motif, Carlsbad, CA) in a 96 well-plate format. Briefly, equal amounts of nuclear extracts (2 μg for cell culture nuclear extracts, 6-10 μg for tissue extracts, depending on origin) from each sample were allowed to bind to immobilized oligonucleotides containing the consensus NF-κB binding sequence. In parallel reactions, supplied extracts of Jurkat T lymphocytes (stimulated with phorbol ester and calcium ionophore) were incubated as a positive control for each assay. In some wells, competitive binding experiments were performed by co-incubating the nuclear extracts an excess (20 pmol) of wild-type or mutated oligonucleotides. Samples and controls were incubated at room temperature for 1 hr on a rotary shaker to ensure complete binding of NF-κB to its consensus sequence. The quantity of bound p65 in each well was detected by incubation with anti-p65 primary antibodies, followed by horseradish peroxidase-conjugated secondary antibodies, and the background signal (obtained by incubation of reagents in the absence of nuclear extracts) was then subtracted from all other values. Chemiluminescence was detected using a Wallac 1420 multilabel counter (PerkinElmer, Shelton, CT). The lower limit of detection for this assay was 0.5 μg of nuclear extract per well. The intra-assay coefficient of variation was <10%, while the inter-assay coefficient of variation ranged from 10-20%. Due to the large between-assay variation, all samples used for direct statistical comparison were run in the same assay.

### Immunofluorescence

Amnion mesenchymal cells were plated onto sterile glass coverslips placed in 4-well tissue culture plates and treated with either IL-1β (10 ng/ml) or TNF-α (20 ng/ml) for 5, 15, 30, 60 and 120 min. Control cells were treated with serum-free medium only. Following each treatment, cells were washed and fixed in 4% paraformaldehyde/PBS solution for 1 h, permeabilized with a 0.2% Triton X-100/PBS solution for 15 min, and blocked in 5% normal goat serum/PBS solution for 1 h. Antibodies directed against NF-κB p65 were applied overnight at 4°C. After washing in PBS, coverslips were exposed to fluorochrome-conjugated secondary antibodies for 1 h at room temperature. Coverslips were then mounted using the ProLong gold antifade reagent with 4',6-diamidino-2-phenylindole (DAPI; Molecular Probes/Invitrogen, Carlsbad, CA) and visualized using an epifluorescence microscope (Zeiss Axiovert 200 M).

To assess the extent of nuclear p65 immunolabeling in the AMC cultures, digital images were acquired from 10 randomly selected, non-overlapping fields from each treatment group. The p65 nuclear labeling index (NLI, defined as the ratio of the number of p65-immunolabeled nuclei divided by the total number of nuclei) was then calculated for each time point.

### Immunohistochemistry (IHC)

Tissues fixed in 10% neutral-buffered formalin were dehydrated and embedded in paraffin. Standard paraffin sections (5 μm) of full-thickness fetal membranes rolls and myometrial biopsies were deparaffinized in xylene and rehydrated through graded ethanol. Immunostaining was performed on using a Discovery autostainer (Ventana Medical Systems Inc., Tucson, AZ). Following antigen retrieval in citrate buffer (pH 6.0), the sections were incubated with 1:1,000-diluted rabbit polyclonal anti-human NF-κB p65 antibody (ab7970, Abcam, Cambridge, MA) for 1 h. Pre-diluted biotinylated universal anti-rabbit/mouse IgG (Ventana Medical Systems Inc., Tucson, AZ) was used as a secondary antibody, and a DAB MAP kit (Ventana Medical Systems Inc., Tucson, AZ, USA) was used for the chromogen reaction. Counter staining was performed with Mayer's hematoxylin. The specificity controls used were a polyclonal rabbit immunoglobulin preparation (product no. 08-6199; Zymed/Invitrogen, Carlsbad, CA) and primary antibody omission control. Additional immunolabeling was performed using primary antibodies raised against p65 phosphorylated at serine 276 (ab2615, Abcam) and p65 phosphorylated at serine 536 (antibody #3031, Cell Signaling Technology, Danvers, MA); however, despite several attempts using a range of titers and antigen-retrieval conditions, we were unable to detect immunolabeling that was distinct from that of controls using these reagents.

A systematic assessment of nuclear p65 immunolabeling in fetal membranes and maternal decidua was performed using a targeted strategy. To this end, an "X" was lightly outlined over each fixed full-thickness fetal membrane roll, and for each specimen, five points of intersection between the "X" and the membrane roll were selected for imaging. Color digital micrographs were obtained using a coded scheme and subsequently analyzed independently by two blinded observers (S.V. and A.A.). The NLI (described above) was then calculated for each image in the amnion, chorion, and decidual layers. The total NLI was calculated as the total number of p65 labeled nuclei in each of the five micrographs for a given layer divided by the total number of nuclei in that layer. Discrepancies were resolved by reanalysis by a single blinded observer (W.E.A.).

For the assessment of the extent of nuclear p65 immunolabeling in myocytes within myometrial biopsy specimens, nuclear p65 immunolabeling was assessed by a single blinded observer (C.J.K.) using a graded scale: no nuclear p65 immunoreactivity (-); isolated nuclear p65 immunoreactivity in a minority of myometrial cells (+); and diffuse nuclear p65 immunoreactivity in a majority of myometrial cells (++).

### Statistical analysis

Statistical analyses were performed using Stata/IC version 10.0 (StataCorp, College Station, TX) and GraphPad Prism version 5.01 (GraphPad Software, La Jolla, CA) software packages. The Shapiro-Wilk test for normality was initially performed on all variables under consideration (demographic data, nuclear labeling indices, and p65 binding activities) to determine whether parametric statistical testing would be valid in each instance. One-way ANOVA was used to compare fetal membrane NLIs in each clinical cohort. For pairwise comparisons of DNA binding activity (e.g., between treated and untreated groups in explants cultures), Student's t-test was employed. For myometrial specimens, the proportions of cases from each cohort exhibiting absent, isolated, or diffuse nuclear p65 immunoreactivity were compared using the Chi-square test. To compare demographic and clinical characteristics between subgroups of the term and preterm cohorts, Student's t-test was also used for parametric continuous variables (maternal age, gestational age at delivery, and birthweight), and Fisher's exact test was used for categorical variables (gravidity and parity).

## Results

### Cytokine stimulation elicits cytoplasmic-to-nuclear translocation of NF-κB p65 in AMC cultures

**Figure 1:**
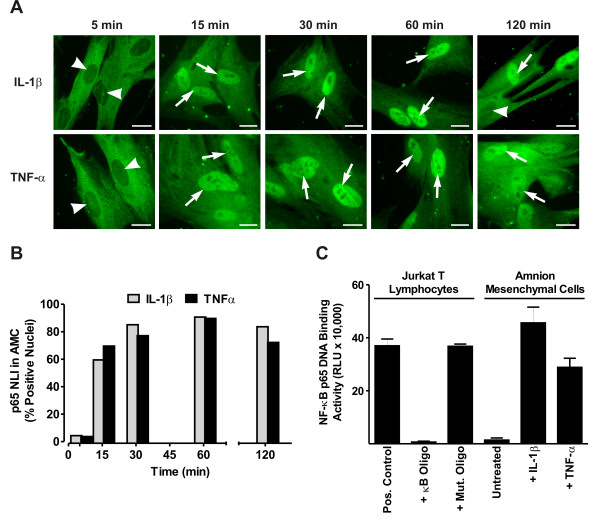
**Correlation between nuclear translocation and DNA binding of NF-κB p65 in AMC cultures**. (A) Intracellular localization of the p65 subunit of NF-κB as detected by immunofluorescence in primary cultures of AMCs treated with 10 ng/ml of IL-1β or 20 ng/ml of TNF-α for 5-120 min. Arrows indicate prominent p65 immunolabeling in cell nuclei, while arrowheads indicate nuclei with deficient p65 labeling. Control cultures exhibited labeling similar to that shown for the 5 min time points (not shown). Bars = 20 μm. (B) Graphical representation of the p65 NLI, depicted as the percent of nuclei with prominent p65 labeling, in AMCs following cytokine stimulation. For each time point, p65 labeling in a minimum of 100 nuclei were enumerated from 10 randomly selected, non-overlapping microscopic fields. (C) DNA binding activity of NF-κB p65 as assessed in nuclear extracts (2 μg/well) of AMCs treated for 30 min with medium alone (untreated control), 10 ng/ml of IL-1β, or 20 ng/ml of TNF-α using an ELISA-based chemiluminescent assay (mean ± SD, N = 3 replicates in a single experiment that is representative of 4 identical experiments obtained using three separate AMC preparations). Values from the luminometer are expressed as relative light units (RLU). As a control for assay specificity, Jurkat T lymphocyte extracts were incubated in the absence (pos. control) or presence of an excess of unbound oligonucleotides bearing either the consensus (κB oligo) or mutated (mut. oligo) NF-κB binding motif.

Studies in our laboratory and by other groups, using many cell culture models, have shown that stimulation with one of many proinflammatory cytokines or bacterial products (e.g., IL-1β, TNF-α, or lipopolysaccharide) promotes the rapid translocation of NF-κB from the cytoplasm into the nucleus, where it acts as a critical transcription factor driving inflammation. In the present work, we examined whether cytokines found in amniotic fluid from women in preterm or term labor could activate cytoplasmic-to-nuclear translocation of the transcriptionally active NF-κB p65 protein subunit in amnion mesenchymal cells grown *in vitro*. Following treatment with either IL-1β (10 ng/ml) or TNF-α (20 ng/ml), there was a time-dependent increase in the loss of cytoplasmic p65 immunoreactivity and a subsequent increase in the accumulation of p65 in the nucleus (Fig. 1A). By calculating the p65 NLI, we observed that maximal levels of nuclear p65 immunoreactivity were reached 30 min after treatment and remained elevated through 120 min (Fig. 1B). Cells treated with medium alone during this time did not exhibit nuclear accumulation of p65 (data not shown).

### Cytokine treatment induces specific binding to the κB oligonucleotide sequence in AMC cultures

We next determined whether cytokine exposure of AMCs could induce the specific binding of cellular proteins to the consensus κB binding element using an ELISA-based assay system. Like the electrophoretic mobility shift assay (EMSA), this assay detects specific DNA-protein interactions, but is designed to enable high throughput and straightforward quantification. Cells were grown for 2 days *in vitro *and the challenged with IL-1β (10 ng/ml) or TNF-α (20 ng/ml) for 30 min followed by nuclear extraction. Nuclear extracts were incubated with the κB oligonucleotide in solid-phase, and the amount specific binding of p65 to the κB binding motif was detected using an antibody and chemiluminescence. We observed a robust increase in binding following either IL-1β challenge (ranging from 5-fold to 33-fold in 4 experiments) and TNF-α stimulation (ranging from 4-fold to 21-fold in 4 experiments), compared to medium-treated control cells (Fig. 1C). Specificity controls revealed that this binding activity could be blocked using an excess of soluble oligonucleotides bearing the κB motif, but not with oligonucleotides bearing the same sequence when scrambled. These results, when combined with the nuclear translocation experiments, indicate that cytokine stimulation leads to the accumulation of nuclear NF-κB which is capable of specific DNA-binding to the κB element.

### NF-κB p65 DNA binding activity in fetal membrane explants and tissues

**Figure 2:**
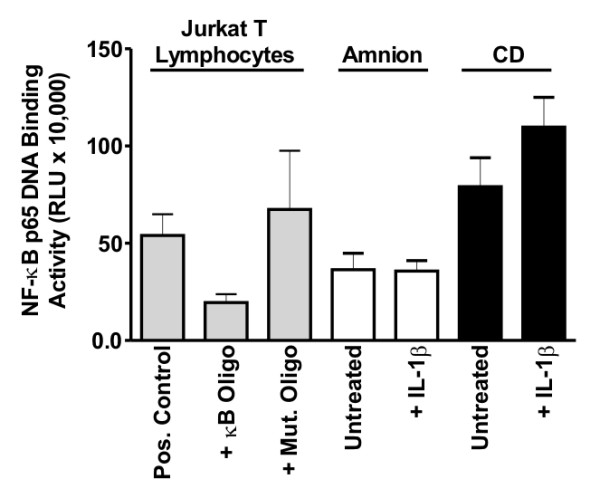
**DNA binding of NF-κB p65 in fetal membrane explant cultures**. Nuclear protein extracts from amnion (10 μg/well) and choriodecidua (6 μg/well) explants incubated in the absence or presence of 10 ng/ml of IL-1β for 2 h were analyzed for NF-κB p65 binding activity using an ELISA-based chemiluminescent assay (mean ± SD, N = 4 explants). Values from the luminometer are expressed as relative light units (RLU). Specificity controls were conducted using Jurkat T lymphocyte extracts incubated in the absence (pos. control) or presence of an excess of unbound oligonucleotides bearing either the consensus (κB oligo) or mutated (mut. oligo) NF-κB binding motif; similar results were obtained when amnion or choriodecidual lysates were incubated with these oligonucleotides (not shown). Relative to the untreated explants, explants stimulated with IL-1β did not show significant increases in p65 binding activity.

Having established that p65 nuclear localization corresponded with its DNA binding activity in AMC cultures, we next sought to extend these results to the more complex scenario of fetal membrane explants. In pilot experiments, we found that p65 DNA binding activity could be recovered at similar levels using either homogenization of unfrozen tissues, or following flash-freezing prior to nuclear extraction. We found that the p65 binding activity was unexpectedly high in unstimulated tissues when compared with untreated AMCs (compare untreated amnion and choriodecidua in Fig. 2 with untreated AMCs in Fig. 1C). In the amnion, there was no increase in binding activity following stimulation with IL-1β for 2 h. While a modest 1.4-fold increase in p65 binding activity was observed in the choriodecidua following IL-1β challenge, this was an order of magnitude less than the increase observed in IL-1β treated AMC extracts, and was not statistically significant.

We next attempted to apply the ELISA-based p65 binding assay to a subset (N = 10/group) of flash-frozen tissue samples (amnion, choriodecidua, and myometrium) in the absence or presence of term labor. In contrast to the results obtained in our IHC analysis (below), we observed no significant differences in p65 binding activity between the TNL and STL groups for any of the tissues examined (Additional File 1: Figure S1). In addition to these experiments, a subset (N = 4) of preterm amnion and choriodecidual specimens were analyzed for p65 binding activity. When these were compared with the binding activities recovered from amnion and choriodecidual samples of the TNL and STL groups, no significant differences were observed (not shown). However, the binding activity in all cases appeared to be specific, since it could be suppressed using an excess of unbound oligonucleotides containing the κB binding motif, but not with oligonucleotides harboring the same sequence when scrambled (Additional File 1: Figure S1). Based on these negative results, we opted not to extend these studies to the remaining tissues collected from these cohorts.

### Immunohistochemical localization of NF-κB p65 in intrauterine tissues in term and preterm parturition

Inasmuch as we were unable to reliably quantify the extent of DNA binding activity of nuclear p65 within frozen tissue samples, a surrogate approach was adopted in which the degree of nuclear p65 immunostaining was evaluated in the fetal membranes, decidua, and myometrium. Such an approach has been employed previously in clinicopathologic studies [20-22] to assess the potential for p65 transactivation based on its cytoplasmic-to-nuclear translocation within tissue specimens, including fetal membranes [23].

**Figure 3:**
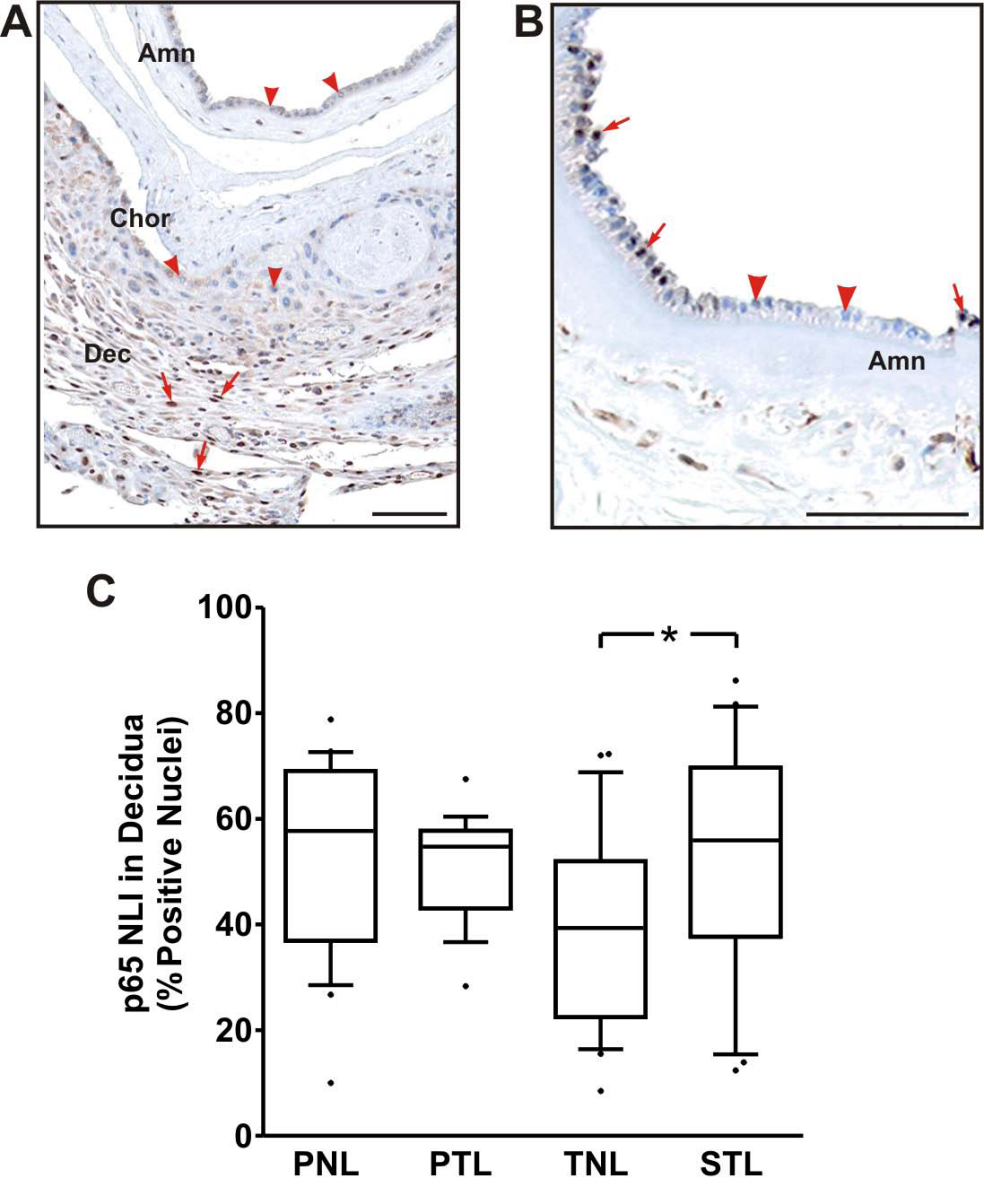
**Immunohistochemical localization of NF-κB p65 in term and preterm fetal membranes and decidua**. (A) Representative photomicrograph demonstrating characteristic p65 immunolabeling. In almost all cases, nuclear p65 labeling was absent in the amnion (Amn) and chorion (Chor), but prominent in the decidua (Dec). (B) Foci of nuclear p65 labeling were exceedingly rare in the amnion and absent in the chorion among all tissues examined. In both panels, arrows indicate areas of with nuclear p65 immunolabeling, while arrowheads demonstrate nuclei with deficient p65 labeling. Bars = 100 μm. (C) Box-and-whiskers plot of the p65 NLI in decidua from the following cohorts: preterm no labor (PNL, N = 22), spontaneous preterm labor (PTL, N = 21), term no labor (TNL, N = 23), and spontaneous term labor (STL, N = 21). NLI was calculated from a total of five micrographs per case, as selected using the targeted strategy described in the Methods section. The NLI in the TNL group was significantly less than that in the STL group (* P < 0.05, one-way ANOVA with Tukey's multiple comparison *post hoc *test).

We found that, with the exception of rare instances (Fig. 3B), nuclear p65 immunolabeling was essentially absent in the amnion and chorion layers, irrespective of clinical context (Fig. 3A). These results were unchanged when we extended our analysis beyond the five photomicrographs targeted for NLI quantification, suggesting that our findings were applicable to the entire fetal-membrane tissues available for analysis. In contrast to the amniochorion, p65 labeling was prominent in decidual layers in all specimens examined. Analysis of the p65 NLI in the decidua revealed that the median NLI was 39.4% TNL cohort (Fig. 3C). Relative to the TNL cohort, the p65 decidual NLI in the TIL group (median: 55.9%) was significantly increased (p < 0.05). The median decidual p65 NLI in the PTL cohort was similar to that of the term labor group (54.7%). Unexpectedly, pronounced nuclear p65 labeling (median: 57.7%) was observed in the PNL group.

**Figure 4:**
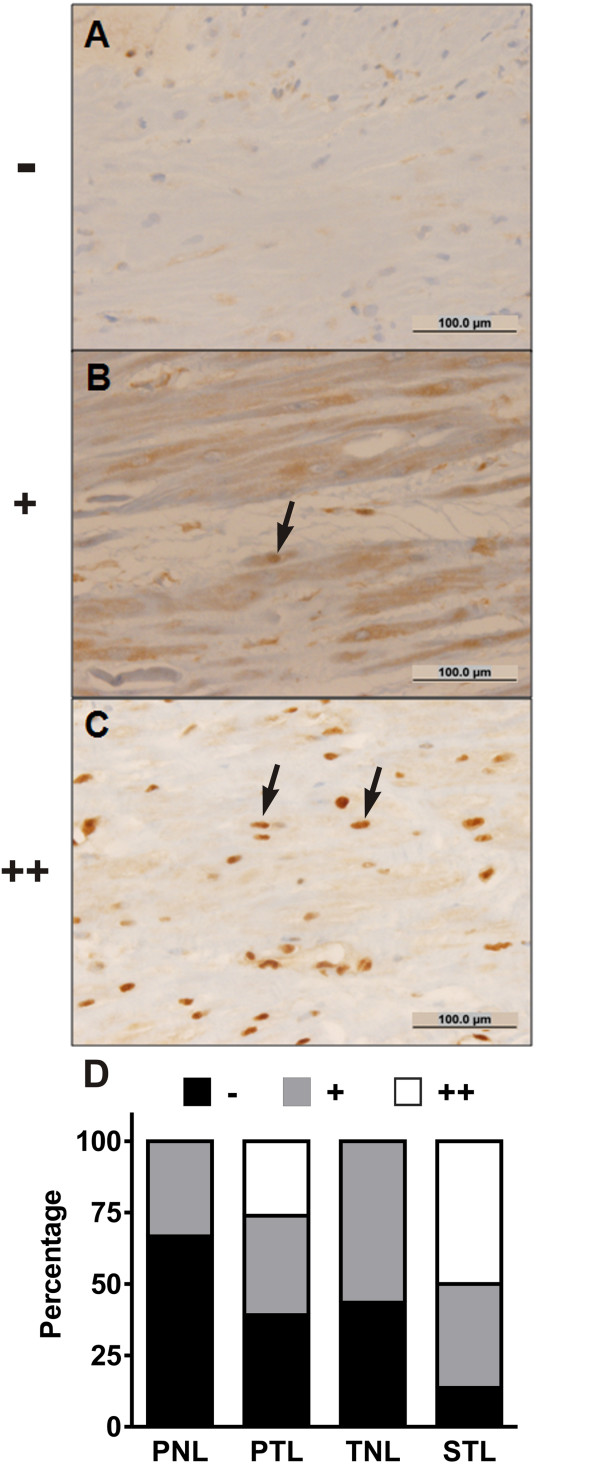
**Immunohistochemical localization of NF-κB p65 in term and preterm myometrium**. (A-C) Representative photomicrographs demonstrating the graded scale used to semi-quantitatively assess the extent of p65 immunolabeling in myocytes: (A) no nuclear p65 immunoreactivity (-); (B) isolated nuclear p65 immunoreactivity in a minority of myometrial cells (+); (C) diffuse nuclear p65 immunoreactivity in a majority of myometrial cells (++). The arrows indicate typical myocytes with nuclear p65 immunolabeling. Bars = 100 μm. (D) Percentage of cases with absent, isolated, and diffuse nuclear p65 staining in myometrial myocytes from biopsy specimens obtained from the following cohorts: preterm no labor (PNL, N = 21), spontaneous preterm labor (PTL, N = 21), term no labor (TNL, N = 23), and spontaneous term labor (STL, N = 21). There were significantly greater proportions of cases exhibiting diffuse nuclear p65 immunolabeling in the PTL and STL groups relative to the unlabored cohorts (P < 0.001, Chi-square test).

In the myometrium, diffuse p65 nuclear labeling (++) in myocytes was observed only in the PTL and STL groups, representing 26% and 50% of cases, respectively (Fig. 4, P < 0.001); no diffuse p65 immunolabeling was observed in the unlabored groups. Correspondingly, the proportion of cases with negative (-) nuclear p65 immunolabeling decreased following preterm or tem labor. While the proportion of cases exhibiting sporadic (+) nuclear p65 labeling was similar between the PNL, PTL, and PNL cohorts (33% to 36%) this proportion was relatively increased in the TNL group (57%).

## Discussion

There is compelling evidence that term and preterm labor are both associated with localized inflammatory responses. As an example, transcriptional profiling has recently demonstrated upregulation in a panoply of immune response genes within full-thickness fetal membranes following spontaneous term parturition [24]. Microarray studies have further suggested that global increases in inflammatory gene expression are more pronounced in the setting of preterm labor, particularly when complicated by intrauterine infection [7]. The NF-κB transcription factors, which govern the regulation of a number of immune response genes, are now thought to play a central role in human labor (see references 25-28 for recent reviews). While highly plausible, the evidence for intrauterine NF-κB activation in term human labor is, in large part, extrapolated from studies using cell cultures *in vitro*. To date, studies of NF-κB activation in tissue specimens have produced inconsistent results. Such inconsistencies provided motivation for the current study, which was developed as a more comprehensive analysis of intrauterine NF-κB activation within intrauterine tissues in the settings of spontaneous preterm and term labor.

Using immunohistochemical analysis, we found that nuclear localization of the p65 NF-κB subunit was essentially absent in the amniochorion, regardless of labor status. While comparable findings have been presented previously [23], these results are in direct conflict with evidence of p65 binding activity based on biochemical analyses of these same tissues. For instance, in pre- and post-labor cultures of amnion cells, EMSA revealed apparent increases in the binding of the p65 and p50 NF-κB subunits to cognate response elements following term labor [29,30]. Similarly, using ELISA-based DNA binding assays, Lappas and Rice have reported that term labor was associated with increased p65 activity in the amnion (but not the choriodecidua) following term labor [27]. In addition, using chromatin immunoprecipitation (ChIP), Mitchell et al. found that p65 binding to the NF-κB responsive IκBα gene promoter (but not the cyclooxygenase-2 promoter) increased in the amnion following term labor [31]. In contrast with these prior reports, we were unable to document biochemical evidence of increased p65 binding activity in either the amniotic or the choriodecidual tissues following term labor.

We have considered that incongruities between the IHC and biochemical results may reflect inherent limitations in one or both of these assays when applied to tissue specimens. Notably, we observed unexpectedly high levels of presumptive p65 DNA binding in amnion, choriodecidua, and full-thickness fetal membrane explants, even in the absence of stimulation. These results are in sharp contrast to those obtained when the same assay was applied to primary cultures of amnion mesenchymal cells, in which there was good agreement between the binding assay and immunolabeling results. Inasmuch as the binding activity from tissue extracts could be competitively diminished using an excess of unbound oligonucleotides containing κB binding elements (but not with oligonucleotides harboring the same sequence when scrambled), and also required p65-like immunoreactivity for detection, there is little reason to believe that such results were due to nonspecific interference with the DNA binding assays. A more likely explanation is that, unlike the cultured cells, enrichment for nuclear p65-containing NF-κB complexes was incomplete during the isolation of nuclei from tissues; thus, significant interference may have resulted from co-extraction of an excess of p65 from extranuclear sources when explants or frozen tissue samples were used. On the other hand, given that a greater amount of tissue was available for query by biochemical analysis relative to IHC, it is also possible that under-sampling may have contributed to an erroneously low estimate of the extent of p65 nuclear labeling by immunolabeling. However, this seems unlikely, given that nuclear p65 labeling was consistently absent in nearly all of the 87 fetal membrane micrographs available for examination.

Unlike the amniochorion, decidual p65 nuclear immunolabeling was evident in all specimens examined. Consistent with numerous reports of increased NF-κB responsive genes in fetal membranes following term labor [5,7,24,25,28], we observed that the decidual p65 NLI was significantly higher in the STL cohort relative to the TNL group. However, since the median decidual p65 NLI was still approximately 40% in TNL specimens, these results also suggest that a lesser degree of NF-κB activity may exist prior to the onset of term labor. Such activity could partially explain the observation that a subset of immune response genes are expressed at high levels in term, unlabored fetal membrane specimens [24].

Unexpectedly, we observed substantial levels of decidual nuclear p65 labeling in both of the preterm cohorts, irrespective of labor status. These results differ from those of Yan and colleagues, who observed a significant increase in the nuclear staining of p65 in term decidua compared to tissues obtained following preterm labor [23]. While p65 nuclear localization is an imperfect surrogate for NF-κB transactivation, our IHC results suggest that decidual cells possess some potential for tonic NF-κB activity; however, considering that such labeling can occur in the absence of preterm labor (as was observed in the PNL group), the significance of this observation may differ from that in the term setting. This could potentially be due to underlying occult inflammation within decidual tissues isolated from preterm, apparently "control" specimens. In addition, it is plausible that other nuclear receptors, such as isoforms of the progesterone receptor [28,32] and peroxisome proliferator-activated receptors (PPARs) [27], contribute to the repression of fulminant NF-κB transactivation in the decidua during the bulk of gestation. Alternatively, given the high prevalence of preeclampsia in the PNL group, we considered that an amplified immune response within specimens delivered of preeclamptic mothers might have skewed our results; however, when the decidual NLI was stratified based on absence or presence of preeclampsia, the labeling indices in each subgroup did not differ significantly from one another, nor from the PTL group (results not shown).

Another possibility for the observed high levels of nuclear NF-κB in non-laboring tissues lies within the heretofore unappreciated biological complexity of the NF-κB transcription pathway, especially with regard to the inhibitor proteins collectively known as IκB. Most studies reported to date in the context of parturition have dealt with the NF-κB and IκB system as something of a binary switch between an active, free NF-κB inflammatory transcription factor and the NF-κB: IκB complex that is transcriptionally silent and sequestered in the cytoplasm [26]. This is an oversimplification. Rather than a simple schema in which NF-κB resides entirely in the cytoplasm under resting conditions, and is then rapidly transported to the nucleus following proinflammatory stimulation, recent studies have demonstrated that p65 complexed to other NF-κB subunits shuttles in and out of the nucleus in an oscillatory pattern [33-35]. Using mathematical modeling, it has been predicted that at any one instant in time, NF-κB could indeed be localized in the nucleus, even in the absence of cell stimulation and, this could be due in part to the complexity of the IκB inhibitors and cell to cell variability [36,37]. Thus, it is highly possible that, during the collection of tissue specimens in both the pre-laboring and post-laboring conditions, immunostaining in one or more tissues could localize p65 within the nuclear compartment, even in the absence of inflammation (i.e., signals heralding labor). These oscillations could also be translated into the finding of elevated DNA binding events even under resting conditions. Therefore, when attempting to consider the global role of NF-κB in regulating parturition in human tissue specimens, some caution is warranted.

In the myometrium, we observed no diffuse nuclear p65 labeling in myometrial cells collected in the absence of preterm or term labor. In comparison, the proportion of cases with diffuse p65 labeling increased following both preterm and term labor, suggesting: (1) that prominent p65 labeling in the decidua can exist without apparent extension to the myometrium (as is evident in paired specimens collected in the PNL cohort); (2) that diffuse nuclear p65 labeling in myometrial smooth muscle cells is specifically associated with labor (indicating that such labeling may correspond with increased NF-κB transcriptional activity); and (3) that labor can occur in the absence of diffuse myometrial p65 labeling, at least in the regions we studied. Regarding the latter point, it is to be noted that all of the specimens available for our analysis were sampled from the lower uterine segment; therefore, we were unable to examine p65 labeling in the uterine fundus. Condon et al., using nuclear and cytosolic extracts collected from a small number of samples from the lower uterine segment and fundal myometrium, observed a pronounced increase in nuclear levels of p65 following term labor only in the fundus [38]. In contrast, Chapman et al. reported low levels of p65 NF-κB DNA binding activity in the lower uterine segment using EMSA and supershift assays [39]. We speculate that diffuse p65 nuclear labeling might have been observed in a greater proportion of post-labor cases had the fundal portion of the myometrium been available for analysis. Nevertheless, given the relatively large number of samples used in the current study, we observed that a proportion of cases (~25% in the preterm cohort, and ~50% in the term cohort) exhibited potential for increased NF-κB transactivation in the lower uterine segment following labor. In this setting, NF-κB activation may promote uterine contractility via upregulation of contraction-associated genes, such as cyclooxygenase-2, oxytocin receptor, and connexin 43 [28].

Strengths of the current study include its size, as well as the availability of paired myometrial and full-thickness fetal membrane specimens for analysis of p65 immunolocalization. While we intended to supplement these results with quantification of NF-κB DNA binding capacity in each sample, as we progressed from cells to explant cultures to tissues, we became less confident in the ability of the assay to accurately reflect physiologically relevant changes in p65 activity. Nevertheless, we believe that the systematic approach presented here illustrates the potential limitations of DNA binding assays when applied to complex tissues. In future studies, the use of a surrogate approach, such as ChIP analysis (which, in theory, should provide a sensitive index of transcription factor binding in the context of specific gene promoters without the need for stringency in nuclear isolation), might be of greater value. Like all studies that rely on the collection of tissues post-delivery, we were limited in the availability of time points for sampling, and were unable to follow changes longitudinally in a given subject. As such, it is possible that transient changes in p65 nuclear localization, as might occur just prior to labor or only in its earliest stages, might have been missed. Furthermore, it is known that p65 is the target of a number of posttranslational modifications, such as phosphorylation and acetylation, which serve to modulate its transactivation potential [40]. Although it would have been of interest to study such modifications in greater detail, attempts to utilize antibodies directed against post-translationally modified p65 were unsuccessful.

In conclusion, the current results suggest that the decidua is an important site of NF-κB regulation in the fetal membranes during the latter part of gestation. Inasmuch as prominent nuclear p65 labeling was observed in the absence of labor or apparent extension to the myometrium, we speculate that mechanisms other than cytoplasmic sequestration must exist to limit fulminant transcriptional activation of NF-κB-responsive genes prior to term.

## Competing interests

The authors declare that they have no competing interests.

## Authors' contributions

SV conducted the transcription factor binding assays using fetal membrane explants and flash frozen tissue samples, in addition to conducting an independent assessment of nuclear immunolabeling in fetal membranes/decidua, and assisting with figure preparation, data analysis, and drafting the manuscript. AA performed the immunohistochemical labeling of tissue sections and conducted an independent assessment of nuclear immunolabeling in fetal membranes/decidua. CJK supervised the immunolabeling assessments performed by SV and AA, and conducted the blinded, semi-quantitative analysis of myometrial immunolabeling. TLSS assisted with immunofluorescence and transcription factor binding assay experiments using amnion mesenchymal cells. JPK participated in the design of the study, facilitated the acquisition of human tissues, and reviewed manuscript drafts. JDI participated in the design of the study and reviewed manuscript drafts. RR participated in the design of the study, facilitated the acquisition of human tissues, and reviewed manuscript drafts. DAK conceived the initial study design, and participated in data analysis, statistical interpretation, and revising the manuscript. WEA participated in the design of the study, supervised all aspects of data collection and analysis, assisted with the *in vitro *experiments, prepared the figures, and participated in the revision of the manuscript. All authors read and approved the final manuscript.

## Supplementary Material

Additional file 1 **Supplemental Figure 1: Effect of labor status at term on NF-κB p65 DNA binding activity**. (A-C) Nuclear protein extracts from amnion (A), choriodecidua (B), and myomterium (C) collected from women before and after term labor and analyzed for NF-κB p65 DNA binding activity using an ELISA-based chemiluminescent assay (mean ± SD, N = 10 tissues/cohort). Values from the luminometer are expressed as relative light units (RLU). There were no statistically significant differences observed between the TNL and STL groups. (D-E) Specificity controls were conducted using amnion, choriodecidua (CD), or myometrial extracts incubated in the absence (no oligo) or presence of an excess of unbound oligonucleotides bearing either the consensus (κB oligo) or mutated (mut. oligo) NF-κB binding motif (mean ± SD of three technical replicates in a single assay). Click here for file
